# Examination of Undesirable Behaviors Displayed by Faculty Members in the Classroom: Perspectives of Pre-Service Teachers

**DOI:** 10.3390/bs16050698

**Published:** 2026-05-02

**Authors:** Burcu Bilir-Koca, Adil Çoruk

**Affiliations:** Department of Educational Sciences, Faculty of Education, Çanakkale Onsekiz Mart University, Çanakkale 17010, Türkiye; burcubilir@comu.edu.tr

**Keywords:** classroom management, faculty members, higher education, pre-service teachers, undesirable behaviors

## Abstract

Effective teaching and learning in classrooms are achievable only through sound classroom management. While positive attitudes and behaviors exhibited by faculty members enhance instructional quality, undesirable behaviors may impede and negatively influence the teaching–learning process. The purpose of this study is to examine the undesirable behaviors displayed by faculty members in classroom settings based on the perspectives of pre-service teachers. The study adopted a phenomenological design and was conducted with 95 pre-service teachers enrolled in the Classroom Management course at the Faculty of Education, Çanakkale Onsekiz Mart University. Data were collected through a semi-structured interview form and analyzed using content analysis. The findings revealed that undesirable faculty members’ behaviors were characterized as actions stemming from instructors’ inadequacies that negatively affect students and the overall educational process. These behaviors were categorized under four themes: instructional management, time management, communication management, and behavior management. The results indicated that undesirable behaviors predominantly originate from instructor-related factors. Pre-service teachers reported experiencing such behaviors most frequently within the theme of behavior management. These behaviors primarily diminish their motivation and negatively influence their participation and academic performance. Pre-service teachers emphasized the need for both institutional and individual measures to prevent undesirable faculty members’ behaviors.

## 1. Introduction

Classrooms are living and learning environments in which teachers and students spend a significant portion of their time (Turan, 2024), and they function as complex communities characterized by continuous interaction. The behaviors exhibited by teachers, who serve as the leaders of these communities, substantially influence not only teacher–student interactions but also students’ interactions with one another, thereby shaping the academic and social development of the classroom group (Ratcliff et al., 2010). Education inherently involves activities aimed at fostering behavioral change. Throughout this process, teachers are expected to function as role models for students while attempting to bring about desired changes in student behavior. In other words, while teachers may expect students to display certain positive behaviors, they themselves should refrain from demonstrating undesirable behaviors (Demirtaş, 2016).

Ensuring that teaching and learning processes are carried out in alignment with instructional goals is possible only through effective classroom management (Çoban, 2015). Classroom management knowledge and skills are of great importance not only for teachers working in primary and secondary education, but also for faculty members in higher education (N. Özer & Bozanoğlu, 2016). In higher education, the quality of courses is significantly influenced by instructors’ classroom management competencies, their personal and professional development, and the attitudes and behaviors they display in the classroom (A. Boz, 2020). For educational activities to proceed as intended, an appropriate classroom environment must first be established (Gürsel et al., 2004). While positive attitudes and behaviors demonstrated by instructors contribute positively to student learning outcomes, negative attitudes and behaviors often lead to the emergence of undesirable student behaviors in the classroom (Aksu et al., 2008; A. Boz, 2020).

Classroom management is a multifaceted and complex construct (Martin & Sass, 2010). It encompasses various interrelated dimensions, including the management of the physical environment, instructional processes, and time (Khaliq et al., 2025). In the literature, classroom management is generally conceptualized as a multidimensional structure involving instructional organization, time management, communication processes, and behavior regulation (Evertson & Weinstein, 2006; Emmer & Sabornie, 2015). Core practices such as organizing the physical environment, planning and implementing instructional activities, and managing student behavior are considered essential components of effective classroom management (Carter & Doyle, 2006; Levin & Nolan, 2014; C. Weinstein & Novodvorsky, 2011; C. S. Weinstein et al., 2011).

Building on these perspectives, these dimensions are regarded as fundamental to creating effective learning environments, as they shape both teaching practices and student experiences. In line with these frameworks, the themes identified in the present study—namely instructional management, time management, communication management, and behavior management—can be interpreted as reflecting core dimensions of classroom management. Although these themes emerged inductively from participants’ experiences, they are consistent with established theoretical models that emphasize structured instruction, effective time use, clear communication, and the regulation of classroom behavior. Therefore, the findings of this study not only represent participants’ lived experiences but also align with the broader conceptual structure of classroom management, thereby strengthening the theoretical grounding of the research. This conceptual alignment provides a theoretical basis for the categorization of findings and helps explain why these four dimensions emerged as central in organizing participants’ experiences.

In instructional settings, alongside positive attitudes and behaviors, undesirable behaviors may also occur. Such behaviors can be perceived in various ways depending on societal norms, personal characteristics, and individual judgments (Dönmez & Cömert, 2009; Karakaş, 2005). Behaviors that hinder all educational efforts in schools and classrooms, impede the establishment and maintenance of an effective teaching–learning process, and disrupt the functioning of the school are considered undesirable behaviors (Akçadağ, 2023; Başar, 2011; İ. Boz, 2003; Celep, 2008; Çelik, 2003; Çoban, 2015; Küçükahmet, 2001). A review of the literature shows that factors influencing student behavior are typically categorized as in-class and out-of-class factors (A. Aydın, 2014; Başar, 2011; Kadıoğlu Ateş & Gürdağ, 2017; Yiğit, 2004). Out-of-class factors include the family, social environment, and school (Atıcı, 2019; Başar, 2011), whereas in-class factors include classroom structure and physical arrangements, curricula, instructional methods, and characteristics of students and teachers (Kadıoğlu Ateş & Gürdağ, 2017; Mazlum, 2024). Undesirable behaviors may arise due to multiple influences such as students, teachers, the school environment, and classroom structure. Some researchers suggest that these behaviors stem not from students’ personalities but rather from teachers’ actions (Çoban, 2015; Stephens & Crawley, 1994). It is also acknowledged that teachers, like students, may exhibit undesirable behaviors and that these behaviors can affect students (Bonfield, 2003; Dolin, 1995; Toale, 2001). Accurately defining undesirable behaviors in the classroom is essential for selecting appropriate strategies to modify or eliminate them in a healthy and effective manner (A. Aydın, 2014).

Undesirable behaviors impair classroom communication; moreover, the negative attitudes displayed by teachers or faculty members in response to such behaviors may further intensify the problem and complicate the overall classroom environment. A review of the literature on undesirable behaviors reveals that most studies focus on identifying undesirable student behaviors from the perspectives of teachers (Arslan & Aslanargun, 2025; O. Aydın & Baştuğ, 2024; Çetin & Öznacar, 2024; Ekinci & Burgaz, 2009; Gökyer & Doğan, 2016; İnci & Çubukçu, 2020; İş & Abiç, 2024; Korkmaz et al., 2024; B. Özer et al., 2014; Siyez, 2009). Additionally, several studies examine teachers’ reactions to undesirable behaviors (Ada et al., 2005; Anar et al., 2024; O. Aydın & Baştuğ, 2024; Çoban, 2015; Kolak Özdemir & Özdemir, 2023; B. Özer et al., 2014; Siyez, 2009). However, research on undesirable behaviors displayed by teachers and faculty members themselves remains limited (A. Boz, 2020; Erben Keçici et al., 2013; Erkol, 2019; N. Özer & Bozanoğlu, 2016). Yet undesirable behaviors exhibited by teachers and faculty members are considered to negatively influence instructional processes at least as much as undesirable student behaviors (Bolkan & Goodboy, 2013; A. Boz, 2020; Goodboy & Bolkan, 2009; Goodboy et al., 2010; Kelsey et al., 2004; Myers, 2002). Research has shown that undesirable behaviors displayed by faculty members negatively affect students’ learning motivation and hinder effective learning (Frisby et al., 2014; Zhang, 2007). Broeckelman-Post et al. (2015) also found a negative relationship between undesirable instructor behaviors and students’ interest and participation in class. Identifying undesirable behaviors exhibited by teachers and faculty members is crucial for ensuring that the teaching–learning process functions effectively. This is particularly important in faculties of education, which carry the mission of preparing future teachers. Faculty members in such institutions serve as role models for pre-service teachers, making it essential to determine and address their undesirable behaviors. This study aims to examine undesirable faculty members’ behaviors from the perspectives of pre-service teachers. Identifying pre-service teachers’ perceptions of undesirable behaviors, the contexts in which these behaviors occur, and their effects on students is expected to contribute to a more effective and higher-quality instructional process. In this regard, the study seeks to raise awareness about undesirable behaviors displayed by faculty members and to encourage the development of necessary measures to prevent such behaviors in educational settings.

The purpose of this study is to examine the undesirable behaviors displayed by faculty members in the classroom based on the perspectives of pre-service teachers. In line with this purpose, the study seeks to answer the following research questions:

1. How are undesirable behaviors displayed by faculty members in the classroom defined?
2. Which behaviors exhibited by faculty members are perceived as undesirable?
3. Under what circumstances do undesirable faculty members’ behaviors occur?
4. How do undesirable faculty members’ behaviors experienced by pre-service teachers affect their class participation, motivation, and academic achievement?
5. What measures can be taken to minimize undesirable behaviors displayed by faculty members?

## 2. Method

In this study, a phenomenological method—one of the qualitative research methods—was employed. Phenomenology is a qualitative research approach that focuses on evaluating lived experience (Jasper, 1994; Miller, 2003). In a phenomenological study, the primary goal is to describe a universal essence through individuals’ experiences of an event or concept (Creswell, 2007).

Although the data were analyzed using inductive content analysis, the study was framed within a phenomenological design. This is because the primary aim of the research was to explore pre-service teachers’ lived experiences and perceptions regarding undesirable faculty members’ behaviors. In this context, content analysis was employed as a systematic analytical tool to identify patterns and themes within participants’ experiential accounts. Therefore, content analysis served as a means of organizing and interpreting the data, while the overall research design remained phenomenological in nature.

### 2.1. Study Group

The study group consisted of 95 pre-service teachers enrolled at the Faculty of Education at Çanakkale Onsekiz Mart University. The participants were selected using criterion sampling, one of the purposeful sampling methods. Criterion sampling is a technique in which individuals who meet a predetermined criterion set by the researcher are included in the study (Büyüköztürk et al., 2014). The criterion for participation in this study was having taken or currently being enrolled in the Classroom Management course. In order to obtain perspectives from different disciplinary backgrounds within teacher education, participants were intentionally selected from three academic areas: Social Sciences Education, Science Education, and Foreign Languages Education. This diversity was intended to ensure that pre-service teachers from different instructional contexts were represented in the study.

Among the participants, 50 were female (52.63%) and 45 were male (47.37%). The participants represented different academic areas within the faculty: 35 participants (36.84%) were from the Social Sciences Education area, 30 (31.58%) from the Science Education area, and 30 (31.58%) from the Foreign Languages Education area. Detailed demographic characteristics of the participants are presented in Table 1.

### 2.2. Data Collection Tool

The data were collected using a semi-structured interview form consisting of open-ended questions prepared by the researchers. Semi-structured interviews are a data collection technique that enables the collection of in-depth data in line with predetermined themes and allows participants to express their personal experiences in detail (Merriam & Tisdell, 2016). The interview form was developed after a review of the relevant literature and was then submitted for expert evaluation. Two academics who had completed their doctorates in the field of educational sciences and had experience in qualitative research examined the interview form, and necessary revisions were made in line with their suggestions. Following this, a pilot study was conducted with 20 students enrolled in the Turkish Language Teaching program in the Faculty of Education. Based on the results of the pilot study, further adjustments were made, and the interview form was finalized by the researchers.

Although phenomenological research typically relies on in-depth, face-to-face interviews, a written format was preferred in order to reach a relatively large number of participants and to capture a broader range of experiences. This approach allowed participants to reflect on their experiences and provide detailed responses in their own time, which may have facilitated more thoughtful and candid expressions. However, it is acknowledged that the use of a written format may have limited the depth of interaction and the opportunity for follow-up questions that are typically possible in face-to-face interviews. This limitation is considered in the interpretation of the findings.

### 2.3. Data Collection Process

Before the data collection process began, ethical approval confirming that the study complied with research ethics principles was obtained from the Ethics Committee of the Graduate Education Institute at Çanakkale Onsekiz Mart University (Approval Date: 18 April 2025; Decision No: 86/143). The data collection process was conducted after receiving this ethical approval. Before the implementation, participants were informed about the purpose and content of the study, and it was clearly stated that the data obtained would be used solely for scientific purposes and that no identifying information would be requested. Prior to the interviews, participants were provided with an informed consent form, and permission for their voluntary participation was obtained.

### 2.4. Data Analysis

An inductive approach to qualitative content analysis was adopted in the analysis of the research data. The primary aim of inductive content analysis is to generate meaningful patterns, concepts, and themes directly from the data without being constrained by a predetermined theoretical framework. Within this approach, codes and themes are derived from participants’ statements, and the analytical process progresses in a data-sensitive and iterative manner (Krippendorff, 2019; Miles et al., 2020). In accordance with the principles of inductive content analysis, themes and codes were developed during the data analysis process. First, all interview transcripts were read by the researchers, and each transcript was examined thoroughly. Common concepts and ideas within the interviews were identified and defined, leading to the development of codes. During the coding process, the researchers first analyzed the data independently and generated initial codes. Subsequently, they compared their coding to identify similarities and differences. Any discrepancies were discussed in detail and resolved through consensus. This iterative process enhanced the consistency and reliability of the analysis. In the second stage, related codes were grouped together to form themes. The compatibility of the codes with the themes was discussed and reviewed by the researchers, and the themes were finalized accordingly. Data saturation was considered during the analysis process. As the analysis progressed, no new themes or categories emerged, indicating that the data reached a point of saturation and provided sufficient depth to understand the phenomenon. The frequency values (f) reported in the findings represent the number of participants who mentioned each code, rather than the total number of occurrences.

### 2.5. Trustworthiness

To ensure inter-coder reliability during the analysis process, the researchers continuously discussed the codes and themes, expressing both agreements and disagreements with one another. To enhance credibility, the researchers frequently compared the results they obtained after analyzing the data. In addition, an academic expert from the Department of Educational Sciences, who specializes in qualitative research and the subject area, was asked to review the study in depth as part of the credibility procedures. To enhance the transferability and consistency of the study, the researchers engaged in ongoing discussions to monitor and control their own potential biases. By openly discussing their assumptions and potential subjective interpretations, they aimed to minimize any researcher bias that could influence the study. To further strengthen internal validity, the data were examined not only by the researchers but also by two additional academics from the Department of Educational Sciences. To ensure reliability, the procedures followed in every stage of the study were described in detail by the researchers. Participants’ direct quotations were included to support the findings and enhance the quality of the study. In addition, triangulation was employed during the data analysis process to enhance the quality of the study. The data were analyzed separately by each researcher, and the results were then compared to reach a consensus. In addition, inter-coder reliability was calculated using the formula proposed by Miles and Huberman (1994) [Reliability = Agreement/(Agreement + Disagreement) × 100]. The agreement coefficient was found to be 96.10%, indicating a high level of consistency between coders.

## 3. Results

In this section, the findings of the study are presented in accordance with the sub-objectives determined for the research.

### 3.1. Definition of Undesirable Faculty Members’ Behaviors

The findings related to pre-service teachers’ views on the definition of undesirable behaviors displayed by faculty members in the classroom are presented in Table 2. These findings indicate that pre-service teachers define undesirable faculty members’ behaviors not only as isolated actions, but also as experiences that influence their emotional engagement, sense of belonging, and trust in the instructional environment.

An examination of Table 2 shows that the definitions of undesirable behaviors displayed by faculty members in the classroom were grouped under three themes: behaviors that negatively affect students, behaviors that negatively affect the teaching–learning process, and behaviors stemming from faculty members’ inadequacies. Within the theme of behaviors that negatively affect students, pre-service teachers defined the undesirable behaviors displayed by faculty members as behaviors that reduce students’ interest and motivation to succeed toward the course, humiliate or embarrass students, or cause them to feel uncomfortable or dissatisfied, and involve unfair or biased behaviors that undermine students’ trust in the instructor. Pre-service teachers most frequently defined the undesirable behaviors exhibited by faculty members in the classroom as “behaviors that reduce students’ interest and motivation to succeed toward the course” (f = 37). Selected quotations from pre-service teachers regarding this theme are presented below.

“Any behavior in the lesson that lowers my morale and reduces my interest and engagement.”

(P25)

“Behaviors that make students uneasy and diminish not only their desire to learn but even their willingness to come to school.”

(P45)

“Disrespectful or belittling behaviors exhibited toward students.”

(P67)

These definitions indicate that such behaviors are experienced as emotionally discouraging and as diminishing students’ engagement and sense of belonging in the learning process.

Within the theme of behaviors that negatively affect the teaching–learning process, pre-service teachers defined undesirable faculty members’ behaviors as actions that reduce or disrupt the quality of instruction, negatively influence communication in the classroom, and hinder effective time management. Pre-service teachers most frequently described undesirable behaviors in this theme as “behaviors that reduce the quality and flow of the teaching-learning process” (f = 29). Selected quotations from pre-service teachers regarding this theme are presented below.

“When I think about this, the undesirable behaviors that come to mind are those that undermine communication.”

(P10)

“These are behaviors within the teaching-learning process that may distance students from the course and even from the instructor.”

(P11)

“Any behavior during the lesson that disrupts the flow, distracts students, or reduces the effectiveness of the class.”

(P39)

These definitions suggest that such behaviors are experienced as disrupting the instructional flow and creating a sense of disconnection between instructors and students.

Within the theme of behaviors stemming from faculty members’ inadequacies, pre-service teachers defined the undesirable behaviors exhibited by faculty members as behaviors reflecting insufficient or inconsistent professional knowledge and classroom practices, performed to assert authority, and unethical or professionally inappropriate. In this theme, pre-service teachers most frequently described undesirable behaviors as “Behaviors reflecting insufficient or inconsistent professional knowledge and classroom practice” (f = 16). Selected quotations from pre-service teachers regarding this theme are presented below.

“I would define it as the instructor’s failure to fulfill their responsibilities.”

(P30)

“Undesirable behaviors displayed by faculty members can be defined as attitudes that negatively affect students’ learning process, reduce motivation in the educational environment, and are unethical.”

(P33)

These definitions indicate that such behaviors are interpreted as signs of professional inadequacy and are associated with a loss of credibility and respect toward the instructor.

### 3.2. Undesirable Behaviors Displayed by Faculty Members

The findings related to pre-service teachers’ views on the undesirable behaviors displayed by faculty members in the classroom are presented in Table 3.

An examination of Table 3 shows that the undesirable behaviors displayed by faculty members in the classroom were grouped under four themes: instructional management, time management, communication management, and behavior management. When the theme of instructional management was examined, pre-service teachers regarded insufficient pedagogical competence, authoritarian, rigid, or ineffective classroom management practices, a lack of instructional preparation and commitment to courses, inconsistent, biased, or intimidating assessment and grading practices, off-topic instructional behaviors, and the inability to motivate students and clearly articulate instructional goals as undesirable faculty members’ behaviors. The behavior most frequently reported by pre-service teachers within this theme was “insufficient pedagogical competence” (f = 27).

Within the theme of time management, pre-service teachers reported several undesirable behaviors displayed by faculty members, including arriving late to or not showing up for class, canceling or interrupting scheduled instructional time, ineffective use of instructional time during the lesson, and failure to manage lesson flow and pacing. The most frequently reported undesirable behavior in this theme was “arriving late to or not showing up for class” (f = 25).

Within the theme of communication management, pre-service teachers considered several behaviors displayed by faculty members to be undesirable, including ignoring students’ feedback and maintaining one-way communication, the lack of effective communication and interaction with students, the use of inappropriate communication styles and tones, the lack of empathy and responsiveness in communication, and interruptive communication behaviors. The most frequently reported undesirable behavior in this theme was “ignoring students’ feedback and maintaining one-way communication” (f = 29).

Within the theme of behavior management, pre-service teachers considered several behaviors displayed by faculty members to be undesirable, including showing favoritism among students; bullying, threatening, and intimidating behaviors; authoritarian, aggressive, and emotionally harsh behaviors; humiliating, belittling, and mocking behaviors toward students; egocentric, disrespectful, and unethical behaviors; behaviors that reduce students’ motivation and emotional well-being; and ignoring students and displaying emotional distance. The most frequently reported undesirable behavior within this theme was “showing favoritism among students” (f = 34). Since pre-service teachers’ views on undesirable behaviors displayed by faculty members often intersected across multiple themes, selected quotations that represent prominent opinions are presented below without theme-based categorization.

“Behaviors such as teaching the course in a strictly one-way manner without valuing students’ questions, failing to inform students despite arriving late to class, being grade-oriented and using grades as a means of pressure on students, having a grading system that lacks consistency, treating the course as unimportant and conducting it superficially, and using a harsh tone toward students.”

(P4)

“Behaviors such as instructors arriving late to class, informing students at the last minute when they will not attend, conducting the lesson with only certain students, expecting university students of a certain age to strictly adhere to attendance requirements, not taking students seriously, and delivering the lecture without engaging in any interaction with students.”

(P10)

“Not providing feedback, coming to class unprepared, and demonstrating ineffective communication and poor time management.”

(P33)

“Displaying disrespectful or belittling attitudes toward students, coming to class unprepared, engaging in biased or unfair grading practices, being intolerant in communication, consistently arriving late, not finishing the lesson on time, preventing student participation, and imposing personal opinions.”

(P36)

“Behaviors such as not effectively delivering the course content, making discouraging remarks that reduce students’ motivation, arriving late to class, belittling students by saying ‘you do not know,’ avoiding eye contact while teaching, not seeking feedback from students, relying on one-way communication, and teaching the lesson solely through lecturing without diversifying instructional materials and techniques.”

(P38)

“Teaching the lesson by reading directly from slides or papers without even looking at the class, discriminating among students, not responding to questions, and speaking to students using insulting language.”

(P39)

These findings indicate that undesirable faculty members’ behaviors are experienced by pre-service teachers not as isolated or theme-specific issues, but as interconnected and overlapping patterns that co-occur within classroom contexts. These patterns are interpreted as shaping students’ overall learning experience, influencing their emotional engagement, sense of value, and their relationship with instructors.

### 3.3. Situations in Which Undesirable Faculty Members’ Behaviors Occur

The findings related to pre-service teachers’ views on the situations in which undesirable behaviors displayed by faculty members occur in the classroom are presented in Table 4.

An examination of Table 4 indicates that the situations in which undesirable behaviors displayed by faculty members occur were grouped under three themes: faculty member-related situations, student-related situations, and other situations. Within the theme of faculty member-related conditions, pre-service teachers reported that the undesirable behaviors exhibited by faculty members emerge in situations involving a lack of communication/interaction or empathy with students, factors stemming from faculty members’ personal characteristics, ineffective planning or management of the course or classroom, perceiving themselves as superior or acting unfairly, adopting a teacher-centered instructional approach, prioritizing personal interests, and possessing insufficient subject matter knowledge or pedagogical competence. Pre-service teachers most frequently stated that undesirable behaviors displayed by faculty members occurred when there was a “lack of communication/interaction or empathy with students” (f = 52). Selected quotations from pre-service teachers regarding this theme are presented below.

“Based on my own experiences, some undesirable behaviors displayed by faculty members seem to stem from their temporarily low mood on a given day. However, others appear to reflect more stable personal characteristics, as these behaviors seem to be consistently exhibited regardless of the situation. In such cases, rather than focusing on specific behaviors, I find myself questioning how such individuals became university instructors in the first place.”

(P6)

“Undesirable behaviors displayed by faculty members tend to emerge when they do not establish strong communication with students, when they do not know their students or make an effort to understand them, when they simply deliver the lecture and leave, and when they prioritize their financial gain over the intrinsic value of teaching.”

(P7)

“Regardless of the classroom context, when problems in the instructor’s personal life increase, negative behaviors displayed in the classroom also tend to increase.”

(P25)

Within the theme of student-related situations, pre-service teachers reported that undesirable behaviors displayed by faculty members emerged when a student expressed a different opinion about the topic during the lesson, did not listen, participate, or give an incorrect answer during the lesson, received a low grade or was right about an issue. In this theme, pre-service teachers most frequently stated that undesirable behaviors occurred when “a student expressed a different opinion about the topic during the lesson” (f = 17). Regarding the theme of other situations, pre-service teachers indicated that undesirable behaviors displayed by faculty members emerged when the physical conditions of the classroom were inadequate. Selected quotations from pre-service teachers related to these themes are presented below.

“Some instructors may display such behaviors from the very beginning of the course; however, I believe that in most cases these undesirable behaviors emerge when instructors realize—particularly during midterm exams or graded presentations—that students have not sufficiently learned the expected content.”

(P4)

“Such behaviors tend to emerge when students express opinions that contradict the instructor’s views, when instructors are firmly convinced that they are right, and when students provide incorrect answers to questions.”

(P32)

“These behaviors are more likely to occur when the class size is large and the physical conditions are inadequate, when instructors struggle to manage discipline, and when interaction with students is limited.”

(P40)

These findings suggest that undesirable faculty members’ behaviors are experienced as context-dependent and shaped by the interaction between instructors, students, and classroom conditions. These situations are interpreted as reflecting underlying tensions in communication, authority, and expectations within the learning environment.

### 3.4. Effects of Experienced Undesirable Faculty Members’ Behaviors on Class Participation, Academic Achievement, and Motivation

Pre-service teachers were asked about the effects of the undesirable faculty members’ behaviors they experienced during their university education on their class participation, academic achievement, and motivation. The findings obtained from their views are presented in Table 5.

An examination of Table 5 indicates that the experienced undesirable behaviors displayed by faculty members have particularly negative effects on class participation and motivation, while their impact on academic achievement is considered to be relatively lower compared to participation and motivation. Regarding the effects on class participation, pre-service teachers reported outcomes such as losing the desire to attend class, decreased participation, passive and silent participation, hesitating to ask questions, feeling discouraged from interaction, falling asleep or mentally disengaging during class, using the full right to absenteeism, as well as perceiving no change in their class participation. Within the theme of effects on class participation, the most frequently reported code was “Losing the desire to attend/decreased participation in class” (f = 27). Selected pre-service teacher statements related to this theme are presented below.

“It affected my class participation significantly. Because an instructor who does not show sufficient care for their course has very limited contributions to offer to the class. As a result, my interest in attending the course decreased, and I now enter exams relying solely on rote memorization.”

(P9)

“In the following classes, I did not participate in any questions asked or situations discussed. I also attended the class without any expectations.”

(P43)

When the responses related to the effects on academic achievement were examined, pre-service teachers reported outcomes such as a negative impact on academic achievement and course grades, feelings of inadequacy or a lack of competence, achievement not being negatively affected, and succeeding out of stubbornness toward the instructor. Within the theme of effects on academic achievement, the most frequently reported code was “Negative impact on academic achievement/course grades” (f = 15). Selected pre-service teacher statements related to this theme are presented below.

“It affected me negatively. Due to both my health condition and the instructor’s attitude, I received very low grades on both the midterm and final exams. Thinking that I would fail the course, I studied for the make-up exam while crying.”

(P16)

“I did not even want to attend that course, so I used my full right to absenteeism, which resulted in passing the course with a low/average grade.”

(P62)

The effects of undesirable faculty members’ behaviors on motivation were reported more frequently by pre-service teachers than their effects on class participation and academic achievement. Decreased motivation/losing interest in the course emerged as the most prominent views.

In addition, responses related to the effects on motivation included a decrease in respect toward the instructor, regretting their department/university choice, considering dropping out/hopelessness, and emotional distress/daily life impact. Some pre-service teachers also stated that these experiences had a positive effect on their motivation. They reported that they attempted to cope with this situation by approaching the course more cautiously and attaching greater importance to it. Within the theme of effects on motivation, the most frequently reported code was “decreased motivation/losing interest” (f = 44). Selected pre-service teacher statements related to this theme are presented below.

“It decreased my motivation. I used all of my absenteeism rights in that course because I did not want to see the instructor.”

(P73)

“It significantly decreased my motivation not only in that instructor’s course but also in other courses. It negatively affected my overall approach to classes.”

(P89)

These findings indicate that undesirable faculty members’ behaviors are experienced by pre-service teachers as having multifaceted and cumulative effects on their class participation, academic achievement, and emotional engagement. These experiences are interpreted as not only reducing participation and motivation, but also negatively influencing academic performance, shaping students’ sense of competence, well-being, and their overall orientation toward the learning process.

### 3.5. Measures to Reduce Undesirable Faculty Members’ Behaviors

Pre-service teachers were asked what measures could be taken to reduce undesirable behaviors displayed by faculty members, and the findings obtained from their views are presented in Table 6.

The measures proposed to reduce undesirable faculty members’ behaviors were grouped under two main themes: institutional measures and individual measures. Within the scope of institutional measures, issues related to monitoring and evaluation emerged as particularly prominent. Additionally, participants highlighted the necessity of taking appropriate actions based on evaluation results, providing faculty members with periodic in-service training, prioritizing pedagogical competence, social skills, and human values in recruitment and appointment processes, promoting supportive, developmental, and positive instructional environments, and establishing structured feedback and communication mechanisms. Among the institutional measures, the most frequently expressed suggestion was that “faculty members should be periodically monitored and evaluated” (f = 34). Some representative views of pre-service teachers regarding institutional measures are presented below:

“I believe that the general comfort of university instructors stems from knowing that they are not monitored. Therefore, I think they should be evaluated annually by both students and fellow instructors.”

(P4)

“Faculty members, just like us pre-service teachers, should receive communication and classroom management training at regular intervals. They should not stop developing themselves because of their position. They should be evaluated by students who continuously receive instruction from them, and necessary measures should be taken based on the evaluation results.”

(P6)

When the views related to the theme of individual measures aimed at reducing undesirable faculty members’ behaviors were examined, prominent views included faculty members’ demonstration of ethical attitudes toward students, the enhancement of communication skills, participation in professional and personal development activities, being approachable in interactions with students, modeling discipline and responsibility, adopting inclusive and student-centered instructional practices, being responsive to feedback mechanisms and students’ needs, and demonstrating effective instructional preparation. Within the theme of individual measures, the most frequently expressed suggestion was that “faculty members should demonstrate respect, fairness, and ethical attitudes toward students” (f = 15). Some representative views of pre-service teachers regarding individual measures are presented below:

“They should do their job with passion, establish closer relationships with students, and most importantly, believe in us.”

(P38)

“Students should be provided with regular feedback. Instructors may be encouraged to develop their empathy skills. If necessary, psychological support should also be provided to help them cope with stress and pressure.”

(P82)

These findings indicate that pre-service teachers do not position themselves as passive recipients of undesirable faculty members’ behaviors, but as active agents who seek both institutional and individual solutions to improve the learning environment. These suggestions are interpreted as reflecting a desire for more supportive, fair, and responsive educational experiences, as well as a need for accountability and professional growth among faculty members.

Taken together, the findings also reveal a consistent pattern across participant groups. Although the inclusion of demographic identifiers (e.g., gender and academic field) allowed for a more context-sensitive examination of the data, no substantial differences were observed in how undesirable faculty members’ behaviors were described or interpreted across these groups. This consistency suggests that pre-service teachers’ experiences of undesirable faculty members’ behaviors are largely shared, regardless of academic field or gender. In this respect, the findings point to the pervasive and common nature of these behaviors within higher education contexts, indicating that such experiences may reflect broader structural and relational dynamics rather than group-specific perceptions.

## 4. Discussion

This study addressed the research questions by examining how undesirable faculty members’ behaviors are defined, experienced, and interpreted by pre-service teachers. Pre-service teachers defined the undesirable behaviors displayed by faculty members in the classroom under three main themes: behaviors that negatively affect students, behaviors that negatively affect the teaching–learning process, and behaviors stemming from faculty members’ inadequacies. Within the first theme, pre-service teachers described undesirable behaviors as those that reduce students’ interest in the course, diminish their motivation to succeed, and undermine their trust in the instructor; behaviors that students find unpleasant, humiliating, disturbing, or discriminatory. Within the second theme, undesirable behaviors were defined as those that reduce the quality of the teaching–learning process, disrupt its flow, and negatively affect communication and time management. In the final theme, pre-service teachers characterized undesirable behaviors as those arising from a lack of professional competence, authoritarian attitudes, unethical practices, behaviors incompatible with the teaching profession, as well as inconsistency and lack of empathy.

Within the framework of pre-service teachers’ views, the undesirable behaviors displayed by faculty members in the classroom can be defined as behaviors stemming from deficiencies in professional competencies that negatively affect both the teaching process and students. According to pre-service teachers, for a behavior exhibited by a faculty member to be regarded as undesirable, it must hinder not only their own learning but also that of their peers’ learning. Pre-service teachers believe that these behaviors arise primarily not from deficiencies in subject-matter knowledge or general culture, but rather from shortcomings in professional (pedagogical) competencies. From their perspective, faculty members tend to display undesirable behaviors in the classroom because they are unable to perform the teaching profession effectively. Consistent with these findings, the literature includes similar studies indicating that faculty members’ professional teaching competencies are insufficient (Murat et al., 2006; N. Özer & Bozanoğlu, 2016; Şen & Erişen, 2002). Therefore, both the perspectives of pre-service teachers and the existing literature indicate that deficiencies in pedagogical knowledge constitute a key underlying factor in the undesirable behaviors exhibited by faculty members in the classroom.

According to pre-service teachers’ views, the undesirable behaviors displayed by faculty members in the classroom were grouped under four themes: instructional management, time management, communication management, and behavior management. Based on these views, undesirable behaviors were most frequently observed within the theme of behavior management, followed respectively by instructional management, communication management, and time management. This finding differs from those reported in previous studies in the literature. For instance, A. Boz (2020) and N. Özer and Bozanoğlu (2016) found that undesirable behaviors displayed by faculty members were more prevalent in the dimension of instructional management. This divergence from the existing literature may be explained by the specific expectations of the participant group as well as by the evolving perceptions of pre-service teachers, who increasingly prioritize interpersonal conduct and behavioral attitudes over the technical dimensions of instructional management.

Faculty members display a wide range of undesirable behaviors while managing the instructional process in the classroom, including using teaching methods that are not aligned with lesson objectives, not valuing the course, coming to class unprepared, digressing from the topic, demonstrating inconsistency in assessment and evaluation, talking on the phone during class, and imposing their own opinions on students. According to pre-service teachers, faculty members also arrive late to class and cancel lessons shortly before they begin. In terms of communication, faculty members either establish one-way communication with students or fail to communicate altogether, and they tend to disregard feedback provided by students. With regard to behavior management in the classroom, faculty members exhibit numerous undesirable behaviors such as discriminating among students, threatening them, humiliating or scolding them, mocking students, constantly frowning, showing a lack of tolerance, and displaying disrespectful attitudes.

These findings are largely consistent with the results of previous studies in the literature. In their study, Vallade and Myers (2014) identified the use of humiliating language toward students and threatening behaviors as undesirable teacher behaviors. Similarly, Erkol (2019) defined undesirable teacher behaviors as threatening, embarrassing, and humiliating students, discriminating among students, using a mobile phone during class, and perceiving oneself as superior to students. In another study, teacher behaviors that made students unhappy were identified as scolding, getting angry, yelling, unfair practices, not conducting the lesson, insulting students, discrimination, and showing a lack of respect for students’ opinions (B. Aydın et al., 2019). Kumral (2009) characterized negative faculty members’ behaviors as communication breakdowns or inadequacies, weaknesses in subject-matter knowledge and pedagogical formation, rigid attitudes, and disrespectful and belittling behaviors toward students. Beyoğlu and Yalçın (2024) likewise identified not adhering to class start and end times, failing to plan lessons or coming to class unprepared, lack of communication, and inability to show empathy as undesirable teacher behaviors. Kearney et al. (1991) examined undesirable faculty members’ behaviors under three dimensions: incompetence, irresponsibility, and offensiveness. Incompetent behaviors include faculty members’ indifference toward their courses and students, as well as practices that cause confusion among students. Irresponsible behaviors involve neglecting the course and students and failing to fulfil professional responsibilities. Offensive behaviors encompass humiliating, rude, and harassing actions. Similarly, Hershberger (2021) examined undesirable faculty members’ behaviors within these three categories and identified comparable behaviors as undesirable. In conclusion, both the findings of the present study and the existing literature suggest that these behaviors—rooted in instructors’ professional and personal inadequacies—affect not only academic achievement but also the democratic and safe learning climate in higher education.

Based on pre-service teachers’ views, it can be said that the undesirable behaviors displayed by faculty members stem primarily from deficiencies in classroom management skills. Disruptions in the core dimensions of classroom management—namely instructional management, time management, communication management, and behavior management—may hinder the creation of an effective learning environment. Therefore, faculty members are expected to possess not only strong academic knowledge but also the classroom management skills necessary to guide the teaching–learning process effectively. In this context, it can be concluded that undesirable behaviors exhibited by faculty members in the classroom reduce the effectiveness of instruction and negatively affect students’ learning. Sidelinger et al. (2011) reported that undesirable faculty members’ behaviors lead students to disengage from the learning process and shift their attention away from the lesson toward peer interaction. Similarly, Goodboy and Bolkan (2009) argued that students learn less when they encounter undesirable behaviors during the learning process. In another study, A. Boz (2020) also emphasized that undesirable behaviors displayed by faculty members negatively affect the learning process. In light of these findings, establishing a high-quality teaching and learning process in higher education requires faculty members to possess not only subject-matter expertise but also strong pedagogical competence across all dimensions of classroom management.

According to pre-service teachers’ views, the situations in which undesirable behaviors displayed by faculty members occur were grouped under three themes: faculty member-related situations, student-related situations, and other situations. Within the first theme, undesirable behaviors were reported to emerge in circumstances such as faculty members perceiving themselves as superior, experiencing low morale, having problems in their personal lives, failing to plan the lesson, lacking communication and empathy with students, and being unable to answer students’ questions. Within the second theme, undesirable behaviors were reported to occur when a student expressed a different opinion about the topic during the lesson, did not listen in class, received a low grade, or was unable to answer or incorrectly answered the instructor’s question. According to the final theme, undesirable behaviors emerged when the physical conditions of the classroom were inadequate.

From the perspective of pre-service teachers, undesirable behaviors displayed by faculty members in the classroom were found to emerge predominantly from faculty member-related situations, followed by student-related situations and other situational factors. Undesirable faculty members’ behaviors may stem not only from instructors’ individual attitudes and behaviors but also from multiple variables such as the nature of communication established with students, level of lesson preparation, problems in personal life, feedback provided by students, and environmental conditions. In this respect, it appears that the emergence of undesirable behaviors is influenced not only by the attitudes of faculty members and students but also by the physical classroom environment, which plays a determining role in shaping classroom dynamics. In conclusion, although environmental and student-related factors play a role, the predominance of undesirable behaviors linked to instructors’ personal and professional preparedness suggests that the primary responsibility for managing these processes lies with the competencies of academic staff.

When the effects of experienced undesirable faculty members’ behaviors on pre-service teachers’ class participation, academic achievement, and motivation are examined, it is evident that their impact is most pronounced in terms of motivation. The findings indicate that undesirable faculty members’ behaviors lead to a decline in pre-service teachers’ motivation, cause them to lose interest in the course, and result in a decrease in respect toward the faculty member. Findings from several studies (Claus et al., 2012; Demirtaş, 2016; Goodboy & Bolkan, 2009; Kelsey et al., 2004) also indicate that instructor-related undesirable behaviors lead to low student motivation. Likewise, Özhan (2024) found that undesirable teacher behaviors negatively affect students’ motivation. In addition to their impact on motivation, undesirable faculty members’ behaviors were also found to have a considerable effect on class participation. Such behaviors reduce pre-service teachers’ enjoyment of attending classes and lead to decreased participation during lessons. In contrast, the effect of undesirable faculty members’ behaviors on academic achievement was found to be more limited compared to their impact on motivation and class participation. Nevertheless, Özhan (2024) also reported that undesirable teacher behaviors negatively affect academic achievement. In conclusion, these findings suggest that undesirable instructor behaviors primarily influence students’ psychological and affective processes—such as motivation and engagement—while declines in academic achievement appear as a secondary outcome of this initial affective disengagement.

Undesirable behaviors originating from instructors lead to the development of negative attitudes and behaviors among students (A. Boz, 2020). Therefore, it is necessary to implement certain measures to eliminate or at least minimize undesirable faculty members’ behaviors. Measures aimed at reducing such behaviors were classified as institutional and individual precautions. Pre-service teachers’ views were found to concentrate primarily on institutional measures, particularly emphasizing the need for monitoring and supervision, as well as the implementation of appropriate actions based on evaluation outcomes. In addition, the findings highlight the importance of providing faculty members with in-service training at regular intervals. With regard to individual measures, the findings underscore the need to improve faculty members’ communication skills and emphasize the importance of receiving psychological support when necessary. In a study based on student perceptions, Duke (1978) reported that teachers’ prejudices toward students, along with their negative attitudes and behaviors, led to the emergence of undesirable student behaviors (as cited in Dönmez & Cömert, 2009). Therefore, in order to prevent the occurrence of undesirable behaviors in classroom settings, faculty members should first develop effective communication skills and refrain from holding prejudices against students. As emphasized by Çoban (2015), maintaining high communication expectations is essential for managing student behavior effectively. In a classroom environment where communication expectations are met and individuals interact with sincerity and openness, teachers are more likely to be accepted by students. In conclusion, these findings suggest that mitigating undesirable behaviors in higher education depends not only on enhancing instructors’ individual awareness and communication skills but also on fostering a robust institutional culture characterized by transparent oversight, continuous professional development, and student-centered feedback mechanisms.

Overall, the findings of this study provide a comprehensive understanding of undesirable faculty members’ behaviors from the perspective of pre-service teachers. In relation to the research questions, the results reveal that these behaviors are defined not only in terms of instructional shortcomings but also through their emotional and relational impact on students. They emerge within complex and context-dependent situations shaped by instructor characteristics, student interactions, and classroom conditions, and they produce multifaceted effects on participation, academic achievement, and motivation. Taken together, these findings suggest that undesirable faculty members’ behaviors should be understood as interconnected patterns of actions that influence both the instructional process and students’ overall learning experiences. In this respect, the study extends existing literature by highlighting the experiential and relational dimensions of faculty members’ behaviors in higher education contexts.

### 4.1. Limitations

This study has several limitations that should be considered when interpreting the findings. First, the study was conducted with pre-service teachers from a single university, which may limit the transferability of the findings to other institutional contexts. Second, the data were based on retrospective self-reports, which may be subject to recall bias and participants’ subjective interpretations. Third, the use of a written data collection method may have limited the depth of responses compared to face-to-face interviews. Fourth, the cross-sectional nature of the study does not allow for the examination of changes in perceptions over time. Finally, given the evaluative nature of the topic, participants’ responses may have been influenced by social desirability bias.

### 4.2. Recommendations

Based on the findings of the study, in-service training programs aimed at improving faculty members’ classroom management skills can be organized in order to reduce undesirable behaviors displayed in the classroom. Within this scope, professional development opportunities supported by practical training can be offered to faculty members on topics such as using effective instructional methods, establishing two-way communication with students, fostering classroom interaction, and managing behavior and time effectively. Considering that students in higher education are adults, training programs grounded in adult education principles (andragogy) may also be provided to faculty members. Higher education institutions can monitor faculty members’ acquisition and maintenance of these skills and develop evaluation and improvement mechanisms within quality assurance systems that specifically address these areas. In addition, to prevent faculty members’ personal problems from being reflected in the classroom environment, institutional psychological counselling and support services can be made available. Students, on the other hand, can be made aware of issues such as class participation, active listening, and respectful communication, thereby contributing to more effective classroom interaction. Finally, improving the physical conditions of learning environments may help prevent undesirable behaviors by enabling both faculty members and students to engage in a more positive classroom atmosphere.

## 5. Conclusions

This study aimed to examine the undesirable behaviors displayed by faculty members in the classroom from the perspectives of pre-service teachers. The findings revealed that these behaviors are multifaceted and primarily stem from instructors’ pedagogical, communicative, and behavioral inadequacies. Undesirable behaviors were systematically categorized under four dimensions of classroom management: instructional management, time management, communication management, and behavior management. Among these, behavior management emerged as the most prominent domain in which undesirable behaviors were observed.

The study contributes to the literature by shifting the focus from student misbehavior to instructor-related undesirable behaviors, particularly within higher education contexts. In doing so, it provides a comprehensive and empirically grounded framework for understanding how faculty members’ behaviors influence the teaching–learning process. The findings highlight that such behaviors not only disrupt instructional quality but also negatively affect students’ motivation, participation, and overall educational experience.

However, several limitations should be acknowledged. The findings of this study should be interpreted with caution due to contextual and methodological constraints, as they are derived from a specific institutional context and rely on participants’ self-reported experiences. Future research could explore undesirable faculty members’ behaviors in different institutional and cultural contexts to enhance the generalizability of findings. In addition, studies employing in-depth interviews or mixed-method designs may provide a more comprehensive understanding of the phenomenon. Longitudinal research could also be conducted to examine how perceptions of undesirable behaviors evolve over time. Furthermore, future studies may investigate the relationship between faculty members’ undesirable behaviors and specific student outcomes, such as academic engagement, well-being, and learning achievement.

## Figures and Tables

**Table 1:** Demographic characteristics of the participants.

Variable		*n*	%
Gender	Female	50	52.63
	Male	45	47.37
	Total	95	100
Area of Study	Social Sciences Education	35	36.84
	Science Education	30	31.58
	Foreign Languages Education	30	31.58
	Total	95	100

Note: The Social Sciences Education area includes Turkish Language Education, Social Studies Education, and Geography Education. The Science Education area includes Mathematics Education and Science Education programs. The Foreign Languages Education area includes English Language Education and Japanese Language Education.

**Table 2:** Definition of undesirable faculty members’ behaviors displayed in the classroom.

Themes	Codes	f
Behaviors Negatively Affecting Students	Behaviors that reduce students’ interest and motivation to succeed toward the course.	37
	Behaviors that humiliate, embarrass, or make students feel uncomfortable or dissatisfied.	36
	Unfair or biased behaviors that undermine students’ trust in the instructor.	12
Behaviors Negatively Affecting the Teaching–Learning Process	Behaviors that reduce the quality and flow of the teaching–learning process.	29
	Behaviors that negatively affect communication in the classroom.	11
	Behaviors that conflict with effective time management.	6
Behaviors Stemming from Faculty Members’ Inadequacies	Behaviors reflecting insufficient or inconsistent professional knowledge and classroom practice.	16
	Behaviors performed to assert authority.	11
	Unethical or professionally inappropriate behaviors.	10

**Table 3:** Undesirable behaviors displayed by faculty members in the classroom.

Themes	Codes	f
Instructional Management	Insufficient pedagogical competence.	27
	Authoritarian, rigid, or ineffective classroom management practices.	26
	Lack of instructional preparation and course commitment.	17
	Inconsistent, biased, or intimidating assessment and grading practices.	9
	Off-topic instructional behaviors.	9
	Failure to motivate students and clarify instructional goals.	3
Time Management	Arriving late to or not showing up for class.	25
	Canceling or interrupting scheduled instructional time.	11
	Ineffective use of instructional time during the lesson.	3
	Failure to manage lesson flow and pacing.	2
Communication Management	Ignoring students’ feedback and maintaining one-way communication.	29
	Lack of effective communication and interaction with students.	28
	Using inappropriate communication styles and tones.	16
	Lack of empathy and responsiveness in communication.	5
	Interruptive communication behaviors.	1
Behavior Management	Showing favoritism among students.	34
	Bullying, threatening, and intimidating behaviors.	33
	Authoritarian, aggressive, and emotionally harsh behaviors.	33
	Humiliating, belittling, and mocking behaviors toward students.	32
	Egocentric, disrespectful, and unethical behaviors.	24
	Reducing students’ motivation and emotional well-being.	12
	Ignoring students and displaying emotional distance.	8

**Table 4:** Situations in which undesirable faculty members’ behaviors occur in the classroom.

Themes	Codes	f
Faculty Member-Related Situations	Lack of communication/interaction or empathy with students.	52
	Situations stemming from personal characteristics.	27
	When failing to plan, value, or manage the course/classroom effectively.	12
	When perceiving oneself as superior or acting unfairly.	10
	When adopting a teacher-centered instructional approach or prioritizing personal interests.	9
	When lacking sufficient subject-matter or instructional knowledge.	7
	When arriving late.	2
Student-Related Situations	When a student expresses a different opinion about the topic during the lesson.	17
	When a student does not listen, participate, or gives an incorrect answer during the lesson.	9
	When a student receives a low grade or is right about an issue.	5
Other Situations	When the physical conditions of the classroom are inadequate.	3

**Table 5:** Effects of undesirable faculty members’ behaviors on students.

Themes	Codes	f
Effects on Class Participation	Losing the desire to attend/decreased participation in class.	27
	Participating passively or silently/not engaging in the lesson.	6
	Hesitating to ask questions/feeling discouraged from interaction.	4
	Falling asleep or mentally disengaging during class.	2
	Using the right to full absenteeism/avoiding class altogether.	2
	No perceived change in class participation.	1
Effects on Academic Achievement	Negative impact on academic achievement/course grades.	15
	Feelings of inadequacy or lack of competence.	4
	Achievement not being negatively affected.	2
	Succeeding out of stubbornness toward the instructor.	1
Effects on Motivation	Decreased motivation/losing interest.	44
	Decreased respect toward the instructor.	10
	Regretting their department/university choice.	5
	Considering dropping out/hopelessness.	4
	Emotional distress/daily life impact.	3
	Approaching the failed course more cautiously and giving it greater importance.	1

**Table 6:** Measures to reduce undesirable faculty members’ behaviors in the classroom.

Themes	Codes	f
Institutional Measures	Faculty members should be periodically monitored and evaluated.	34
	Faculty members should receive regular in-service and professional development training.	19
	Necessary measures should be taken based on evaluation results.	14
	Faculty members should be reminded of their roles, responsibilities, and professional ethics.	5
	Recruitment and appointment should prioritize pedagogical competence, social skills, and human values.	4
	Supportive, developmental, and positive instructional environments should be promoted.	4
	Feedback and communication mechanisms should be established.	2
	Stress factors such as excessive teaching load and research pressure should be balanced.	1
	Salary deductions should be implemented.	1
	An institutional body where students can seek their rights should be established.	1
Individual Measures	Faculty members should demonstrate respect, fairness, and ethical attitudes toward students.	15
	Faculty members’ communication skills should be enhanced.	14
	Faculty members should engage in continuous professional and personal development.	13
	Faculty members should be approachable and supportive in interactions with students.	7
	Faculty members should model discipline, responsibility, and professional conduct.	7
	Faculty members should adopt inclusive, student-centered and motivating teaching practices.	5
	Faculty members should be sensitive to the feedback mechanism and student needs.	4
	Faculty members should demonstrate effective instructional preparation and delivery.	3

## Data Availability

The data supporting the findings of this study are not publicly available due to ethical restrictions but can be obtained from the corresponding author upon reasonable request.
